# Lipid metabolism reprogramming in head and neck cancer

**DOI:** 10.3389/fonc.2023.1271505

**Published:** 2023-10-20

**Authors:** Jinfeng Liang, Lin Li, Limei Li, Xiaoying Zhou, Zhe Zhang, Yi Huang, Xue Xiao

**Affiliations:** ^1^ Department of Otolaryngology-Head and Neck Surgery, First Affiliated Hospital of Guangxi Medical University, Nanning, China; ^2^ Department of Pediatric Dentistry, College & Hospital of Stomatology, Guangxi Medical University, Nanning, China; ^3^ Key Laboratory of Early Prevention and Treatment for Regional High-Frequency Tumor, Guangxi Medical University, Ministry of Education, Nanning, China

**Keywords:** lipid metabolism, reprogramming, head and neck cancer, biomarker, prognosis

## Abstract

Lipid metabolism reprogramming is one of the most prominent metabolic anomalies in cancer, wherein cancer cells undergo dysregulation of lipid metabolism to acquire adequate energy, cell membrane building blocks, as well as signaling molecules essential for cell proliferation, survival, invasion, and metastasis. These adaptations enable cancer cells to effectively respond to challenges posed by the tumor microenvironment, leading to cancer therapy resistance and poor cancer prognosis. Head and neck cancer, ranking as the seventh most prevalent cancer, exhibits numerous abnormalities in lipid metabolism. Nevertheless, the precise role of lipid metabolic rewiring in head and neck cancer remains unclear. In line with the LIPID MAPS Lipid Classification System and cancer risk factors, the present review delves into the dysregulated molecules and pathways participating in the process of lipid uptake, biosynthesis, transportation, and catabolism. We also present an overview of the latest advancements in understanding alterations in lipid metabolism and how they intersect with the carcinogenesis, development, treatment, and prognosis of head and neck cancer. By shedding light on the significance of metabolic therapy, we aspire to improve the overall prognosis and treatment outcomes of head and neck cancer patients.

## 1 Introduction

Reprogramming of lipid metabolism is a critical hallmark of cancer. In recent years, dysregulated lipid metabolism has emerged as a focal point in cancer research (1). Physiologically, lipids play essential roles as energy sources, vital building blocks for cell membranes, as well as first and second messengers in molecular recognition and signaling processes (2, 3). Pathologically, any disturbance in lipid metabolism would significantly impact serious cellular behaviors, such as cell proliferation, differentiation, apoptosis, and motility, ultimately contributing to carcinogenesis and cancer development (3). In addition, rewiring of lipid metabolism impacts the treatment effects and the outcomes of cancer patients (4). Thus, lipid metabolism-related molecules and pathways have been proposed to be novel targets of anti-cancer treatment.

Head and neck cancer (HNC) comprises a group of tumors that predominantly arise from subsites within the oral cavity, oropharynx, hypopharynx, and larynx (5). It ranks as the seventh most common cancer worldwide, with more than 890,000 new cases and 450,000 deaths each year (6). Head and neck squamous cell carcinoma (HNSCC) accounts for approximately 90% of all HNC cases (7). Infection of human papillomavirus (HPV), tobacco use or smoking, and alcohol consumption are the three well-known risk factors for head and neck cancer (6, 8). Despite notable advancements in diagnosis and treatment, the 5-year overall survival rate for head and neck cancer patients with an advanced stage is 50% to 60%, indicating an urgent demand for further investigation into the underlying mechanisms of head and neck cancer carcinogenesis (9).

Lipid metabolism reprogramming is also linked to the carcinogenesis and development of head and neck cancer. Cancer cells actively promote the lipolysis process. And in turn, the various endpoints of lipid metabolism, including the oxidation of fatty acids, the generation of signaling lipids, epigenetic modifications of proteins, synthesis of cell membrane lipid, and other crucial processes, profoundly affect the malignant characteristics of cancer cells. Accumulated studies have described the dysregulated levels or activities of enzymes and molecules associated with lipid metabolism in head and neck cancer. Alterations of lipid profile in head and neck cancer are evident; for instance, there’s an elevation of fatty acid C16:0 and a reduction in total ceramide (10). In addition, overexpression of crucial components in lipid metabolism, such as cluster of differentiation 36 (CD36), fatty acid-binding protein (FABP), acetyl-CoA carboxylase (ACC), and fatty acid synthase (FASN), have been well-documented in head and neck cancer (11–14). Hence, the molecules involved in the lipid metabolism alteration hold potential as novel biomarkers for the treatment and prognosis of head and neck cancer. Nonetheless, the precise role and underlying mechanisms of lipid metabolism in the context of head and neck cancer remain inadequately understood.

**Figure 1:**
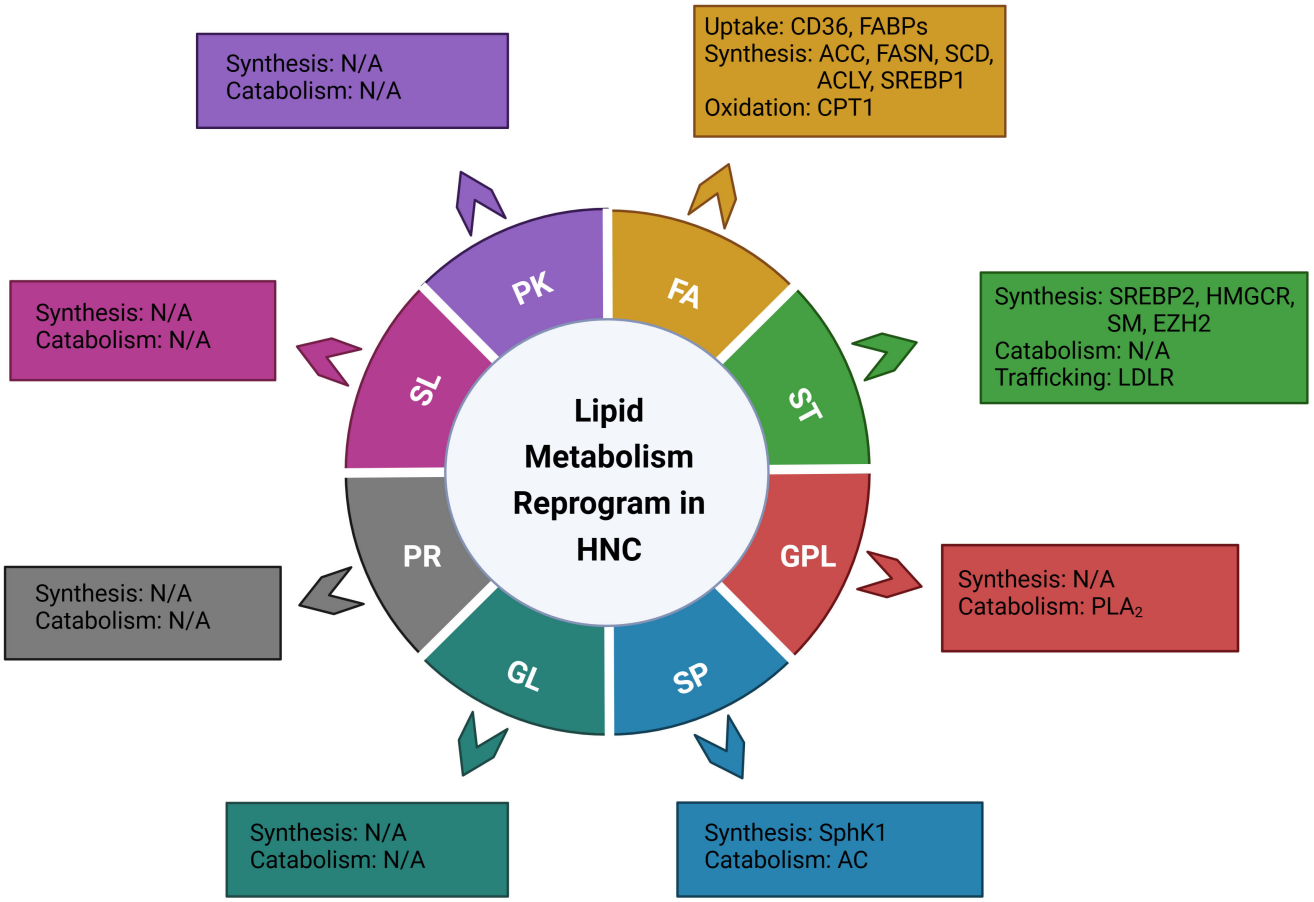
Illustration of the genes or enzymes that have been reported to be involved in the reprograming of lipid metabolism in head and neck cancer, based on the LIPID MAPS Lipid Classification System. FA, fatty Acid; ST, sterol lipids; GPL, glycerophospholipids; SP, sphingolipids; GL, glycerolipids; PR, prenol lipids; SL, saccharolipids; PK, polyketides; CD36, cluster of differentiation 36; FABPs; fatty acid-binding proteins; ACC, acetyl-CoA carboxylase; FASN, fatty acid synthase; SCD, stearoyl-CoA desaturase; ACLY, ATP citrate lyase; SREBP1, sterol regulatory element binding proteins 1; CPT1, Carnitine Palmitoyl Transferase 1; SREBP2, sterol regulatory element-binding protein 2; HMGCR, 3-hydroxy-3-methylglutaryl (HMG)–CoA reductase; SM, Squalene monooxygenase; EZH2, Enhancer of zeste homolog 2; LDLR, low-density lipoprotein receptor; PLA2, phospholipase A2; cPLA2, cytosolic PLA2; sPLA2-IIA, secretory phospholipase A2 IIA; SphK1, Sphingosine kinase 1; AC, Acid ceramidase.

According to the LIPID MAPS Lipid Classification System, lipids are divided into eight categories, known as fatty acids, glycerolipids, glycerophospholipids, sphingolipids, sterol lipids, prenol lipids, saccharolipids, and polyketides (15). In the present review, based on the lipid classification system and cancer risk factors, we focused on the dysregulation of lipid metabolism and its implications on the carcinogenesis, treatment, and prognosis of head and neck cancer. Novel dysregulated molecules and signaling pathways participating in the process of lipid uptake, *de novo* synthesis, transportation, catabolism, as well as their roles in the composition of lipid profiles, would be involved and discussed, aiming to provide an updated and comprehensive understanding of this field and potential strategies for personalized treatment of head and neck cancer (**Figure 1**, **Table 1**).

**Table 1:** Dysregulated lipid metabolism profile in head and neck cancer.

Category	Class or Subclass	Increased in HNC	Decreased in HNC	Cancer type
Fatty acids	Palmitic acid (C16:0)	Dickinson (2020) (16), Halczy-Kowalik (2019) (10)		OSCC
	Oleic acid (C18:1)	Dickinson (2020) (16)		OSCC
	Oleic acid (C18:1n9)	Halczy-Kowalik (2019) (10)		OSCC
	Erucic acid (C22:1n13)	Halczy-Kowalik (2019) (10)		OSCC
	Docosatetraenoic acid (C22:4n6)	Halczy-Kowalik (2019) (10)		OSCC
	Docosapentaenoic acid (C22:5n3)	Halczy-Kowalik (2019) (10)		OSCC
	nervonic acid (C24:1)	Halczy-Kowalik (2019) (10)		OSCC
	FA 14:0		Christou (2021) (17)	HNC
	FA 18:3n-3		Christou (2021) (17)	HNC
	FA 20:3n-6		Christou (2021) (17)	HNC
Glycerolipids	Triglyceride		Poorey (2016) (18), Patel (2004) (19) Somashekar (2011) (20) Garg (2014) (21)	HNC
Glycerophospholipids	Phosphatidylcholine (PC)		Wang (2017) (22)	OSCC
	Phosphatidylcholine (PC)	Dickinson (2020) (16)		OSCC
	Phosphatidylethanolamine (PE)		Wang (2017) (22)	OSCC
	Phosphatidylethanolamine (PE)	Dickinson (2020) (16)		OSCC
	Phosphatidylinositols (PI)	Dickinson (2020) (16)		OSCC
	LysoPC (14:0)		Wang (2017) (22)	OSCC
Sphingolipids	Sphingolipid 42:2		Wang (2022) (23)	LC
	Sphingolipid 42:3		Wang (2022) (23)	LC
	C18-ceramide		Koybasi (2004) (24) Senkal (2010) (25)	HNSCC
	C16-ceramide		Senkal (2010) (25)	HNC
Sterol lipids	Total cholesterol		Garg (2014) (21), Sherubin (2013) (26), Acharya (2016) (27)	OSCC
	Total cholesterol		Pereira (2014) (28)	HNC
	LDL-cholesterol		Pereira (2014) (28)	HNC
	Total cholesterol	Jiang (2021) (29)		HNC
	apoA-I	Huang (2023) (30)		HNC
Prenol lipids	N/A			
Saccharolipids	N/A			
Polyketides	N/A			

FA, fatty acid; OSCC, oral squamous cell carcinoma; HNC, Head and neck cancer; HNSCC, head and neck squamous cell carcinoma; LysoPC, lysophosphatidylcholine; LDL-cholesterol, low-density lipoprotein cholesterol; LC, laryngeal carcinoma; N/A, no report yet.

## 2 Lipid metabolism reprogram in head and neck cancer

### 2.1 Alteration of fatty acid metabolism in head and neck cancer

Fatty acids are composed of saturated or unsaturated hydrocarbon chains terminating with distinct carboxylic acid groups. Mammals acquire fatty acids from the surrounding environment or by synthesizing *de novo* (31). The metabolism of fatty acids encompasses two key biological processes: fatty acid synthesis and fatty acid oxidation (FAO) (32).

#### 2.1.1 *De novo* biosynthesis and catabolism of fatty acids

Fatty acid biosynthesis occurs in the cytosol. The first step involves the conversion of acetyl-CoA into malonyl-CoA through the activation of ACC. This, along with the participation of enzymes such as FASN, leads to the generation of palmitate (FA16:0). Subsequently, elongation processes produce fatty acids of different lengths. These fatty acids can then be esterified with glycerol or sterol skeletons, forming triacylglycerol (TG) or sterol esters, respectively. Finally, they are stored as lipid droplets (LDs) (33). On the other hand, fatty acid catabolism involves the activation of FAs in the cytoplasm to form acyl-CoA. The formed acyl-CoA is then transported to the mitochondrial matrix by carnitine palmitoyltransferase 1 (CPT1) for oxidation (34).

#### 2.1.2 Altered fatty acid profile in head and neck cancer

Dysregulated lipid metabolism in head and neck cancer patients results in a distinctive fatty acid profile. In the context of oral squamous cell carcinoma (OSCC) tissues, it was noted that when compared with healthy controls, the level of fatty acids was frequently elevated, with the most significant increase observed in palmitic acid (C16:0), followed by oleic acid (C18:1) (16). In another study including 30 OSCC patients, Domagala et al. noted a remarkable increase of C16:0 in tumor, adjacent tissue, and blood serum samples. Independent of tumor grade, the levels of oleic acid (C18:1n9), erucic acid (C22:1n13), docosatetraenoic acid (C22:4n6), docosapentaenoic acid (C22:5n3), and nervonic acid (C24:1) were found to be more abundant in tumor-adjacent tissues than in serum, suggesting the potential roles of these fatty acids in promoting tumor progression in OSCC (10). In an explorative study, Laurell et al. compared the circulating lipidomic profile of HNC patients one year before and after treatment. A specific pattern emerged for FA 14:0, 18:3n3, and 20:3n6, showing an early reduction in FA 14:0 and late reductions in FA 18:3n-3 and 20:3n-6. Additionally, FA 14:0 was associated with changes in body weight (17). Taken together, the level of C16:0 is supposed to be increased in head and neck cancer. Nevertheless, a definitive fatty acid profile specific to head and neck cancer remains elusive. To address this, further investigation with a larger sample size and a more rigorous study design is imperative.

#### 2.1.3 Increased fatty acids uptake in head and neck cancer

##### 2.1.3.1 Cluster of differentiation 36

CD36, known as a fatty acid translocase, is responsible for binding and trafficking FAs from the exogenous environment into host cells. Its upregulation has been consistently observed in various human cancers, such as colorectal cancer (35), gastric cancer (36, 37), hepatocellular carcinogenesis (38), and melanoma (39). CD36 plays a vital role in modulating cancer development, metastasis, therapy resistance, and prognosis (40–44). Notably, CD36 is highly expressed in HNC (11, 45–47). Increased expression of CD36 was significantly associated with higher tumor status, tumor grading, and lymph node metastasis rate, exhibiting an over 40-fold increase in oral cancers (45, 46). An intriguing study by Pascual et al. reported that dietary palmitic acid could activate CD36 and accelerate cancer growth in OSCC, suggesting that dietary intake of fatty acids might play a role in CD36 modulation (48). In mice model, inhibition of CD36 has been shown to attenuate the metastasis of several cancers (41, 49, 50), including HNSCC (45). In particular, depletion of CD36 led to a significant inhibition of the lung metastasis ability of OSCC cells (45). Taken together, these findings suggest that CD36 could be a promising therapeutic target for clinical intervention of head and neck cancer.

##### 2.1.3.2 Fatty acid-binding proteins

FABPs are important lipid chaperones, facilitating the transportation of long-chain fatty acids (LCFAs) to specific cell compartments. There are a series of members in the FABP family. The abnormal expression of FABPs in malignant tumors is associated with carcinogenesis, progression, and prognosis (51–55). In the case of HNC, there is notable upregulation of epidermal FABP (E-FABP, also known as FABP5), which might contribute to cancer proliferation and invasiveness (56–58). E-FABP, regulated by the epithelial cell adhesion molecule (EpCAM), is suggested to be a potential target of the oncogene c-Myc, leading to enhanced cell proliferation (57). Interestingly, according to Uma et al., FABP5 exhibited a lower expression in metastatic lymph nodes when compared with the corresponding primary squamous cell carcinoma of the oral tongue tumors (12). Yet, more samples are needed to confirm the conclusion. Furthermore, a study by Ohyama et al. reported abnormal expression of FABP4 and FABP5 in tongue carcinoma. Besides, the cytoplasmic staining for FABP5 was increased in tongue carcinoma patients with advanced T-stage and clinical stage, implicating that FABP5 might be a pathological marker (59). However, the precise mechanisms of FABPs in head and neck cancer need further research and investigation.

#### 2.1.4 Dysregulated fatty acids synthesis in head and neck cancer

##### 2.1.4.1 Acetyl-CoA carboxylases

ACCs serve as rate-limiting enzymes in fatty acid synthesis, playing a crucial role in catalyzing the carboxylation of acetyl-CoA to malonyl-CoA (60). The two isoforms of ACC, namely ACC1 (ACCα) and ACC2 (ACCβ), are enriched in lipogenic and oxidative status, respectively (61). ACCs are highly expressed in several cancer types, including prostate cancer, breast cancer, and HNC (62–64). Notably, elevated expression of ACC2 in laryngeal carcinoma correlates with advanced clinical cancer stage, lower cancer differentiation degree, as well as poor survival rates (64). It is known that ACC is regulated by AMP-activated protein kinase (AMPK) via phosphorylation (65). The same modulation also applies within the context of head and neck cancer (66). HNSCC cells with ACC mutations and lacking the AMPK phosphorylation sites, showed resistance to cetuximab treatment (67). With the combination treatment of ACC inhibitor 5-(tetradecyloxy)-2-furoic acid (TOFA) and cetuximab, the growth of cetuximab-resistant HNSCC xenografts was effectively suppressed, suggesting that ACC can rewrite cancer metabolism from glycolysis-dependent to lipogenesis-dependent way (67). However, Chen et al. reported that an enhanced level of pACC was independently associated with poor overall survival of HNSCC patients, particularly in patients with lymph node-metastasis and advanced stage (13). The potential causes of contradiction may stem from various factors, including different ACC subtypes, diverse confounding factors (e.g., TNM stage, primary tumor site), and variation in experimental systems (e.g., *in vivo*, *in vitro*, or patient-based studies), among others. Additionally, phosphorylation of ACC is not only governed by AMPK; other kinases such as protein kinase C (PKC) and casein kinase 2 (CK2) also serve as regulators (60) Therefore, further evaluation is warranted to explore the modulation of ACC and its role in head and neck cancer.

##### 2.1.4.2 Fatty acid synthase

FASN is a critical enzyme responsible for generating long-chain fatty acids from acetyl-CoA and malonyl-CoA (68). The level of FASN is naturally low or even undetectable in most human tissues except lactating breasts and cyclical endometrium, as the daily requirements of fatty acids are adequately met by food consumption (68, 69). In contrast, high levels of FASN are observed in various malignancies, which have been linked to increased risks of cancer metastasis, recurrence, and poor survival (70–74). In accordance with this, the expression of FASN is obviously elevated in HNSCC (14, 75–77). Salivary gland carcinoma, including well-differentiated carcinoma (secretory carcinoma, acinic cell carcinoma, etc.), high-grade adenoid cystic carcinoma, and mucoepidermoid carcinoma, also show an increased level of FASN, suggesting its potential role as an indicator of cancer aggressiveness and differentiation (78). Upregulation of FASN is required for the proliferation of oral squamous carcinoma (79). Pathological analysis has indicated that FASN expression was correlated with lymphatic infiltration, perineural infiltration, and regional lymph node metastasis status in OSCC (76). Moreover, high expression of FASN was related to poor prognosis and might be an indicator of pulmonary metastasis in patients with HNSCC (76, 77, 80).

FASN is positively regulated by the cell surface receptor ErbB2 in HNSCC (75, 80, 81), though the precise correlation and underlying mechanisms need further investigation. As a FASN inhibitor, orlistat has demonstrated promising effects in inhibiting the proliferation and metastasis of orthotopic tongue oral squamous cell carcinoma (82). Further, *in vivo* studies have shown that orlistat reduces the cervical lymph node metastasis rate by 43% (82). Additionally, orlistat or FASN siRNA treatment increased cell cytotoxicity and cell sensitivity to radiotherapy in the radioresistant HNSCC cell line rSCC-61 (83). Orlistat also increased the chemosensitivity of OSCC cells to cisplatin and paclitaxel by downregulating cyclin B1 (84). Moreover, other FASN inhibitors, such as C75 and Tvb-3166, have been shown potential as antineoplastic agents (84, 85). Further research is needed to explore the therapeutic implications of targeting FASN in HNC treatment.

##### 2.1.4.3 Stearoyl-CoA desaturase

SCD is a lipid-modifying enzyme catalyzing the mono-saturation of oleate (18:1) and palmitoleate (16:1) (86). There are two isoforms of SCD (SCD1 and SCD5) required for human lipid metabolism (87). In a series of human malignancies such as lung, breast, colorectal, and bladder cancers, SCD is found to be overexpressed, and its upregulation is associated with tumor aggressiveness, making it a novel prognostic marker (88–90). In OSCC, elevated expression of SCD was negatively correlated with survival (91). Interestingly, overexpression of SCD was detected not only in HNSCC cell lines but also in tobacco-treated normal oral keratinocytes. Inhibition of SCD hampers cell proliferation, invasion, and colony formation, suggesting the potential roles of SCD and tobacco in the carcinogenesis of HNSCC. Thus, SCD has been proposed as a therapeutic target for HNSCC patients, particularly those with a history of tobacco use (92).

##### 2.1.4.4 ATP citrate lyase

ACLY is a cytosolic enzyme which is responsible for acetyl-CoA synthesis during *de novo* lipogenesis (93). It is frequently upregulated in various malignancies, such as colorectal cancer (94), glioblastoma (95), endometrial cancer (96), and non-small cell lung cancer (97). In HNSCC, ACLY is also upregulated (91, 98) and its heightened expression is associated with an unfavorable prognosis (98). In addition, elevated ACLY expression is associated with the failure of radiotherapy. Notably, treatment of HNSCC cells with the ACLY inhibitor BMS303141 has been demonstrated to facilitate radiosensitivity via disturbing the DNA damage repair process (98), implying the potential role of ACLY inhibitor as a sensitizer for head and neck cancer treatment. Additionally, acetyl-CoA, generated by ACLY, is essential for acetylation reactions, particularly histone acetylation. Thus, ACLY is a critical node which links cellular metabolism to epigenetic modification. In nasopharyngeal carcinoma, ACLY is found to be protected from ubiquitin degradation through its interaction with the long noncoding RNA TINCR, thereby promoting cancer cell proliferation, metastasis, and chemotherapy resistance (99). Nonetheless, there’re limited reports on the role of ACLY in head and neck cancer, warranting further in-depth investigation.

##### 2.1.4.5 Sterol regulatory element binding proteins

SREBPs is a family of transcription factors located in the endoplasmic reticulum. In mammalian cells, three SREBPs (SREBP-1a, -1c, and -2) are expressed, which are encoded by SREBP1 and SREBP2, respectively. Among them, SREBP1 is a major transcriptional regulator of fatty acid synthesis, while SREBP2 is mainly responsible for cholesterol metabolism, which will be discussed later. SREBP1 is highly activated in cancers, and its overexpression is correlated with increased cancer aggressiveness (100–105). It has been reported that overexpression of SREBP1 is necessary for HNSCC cell proliferation and migration (106). Furthermore, SREBP1 is noted to be an important linker between tumor protein p63 (TP63) and fatty acid metabolism, suggesting its potential role as an independent prognostic and therapeutic marker in HNSCC (106). Consistently, Su et al. reported that SREBP1 functions as an oncogene via upregulating steroidogenic acute regulatory protein-related lipid transfer 4 (STARD4) and promoting immune cell infiltration in HNSCC (107). Furthermore, SREBP1-mediated cell survival is disturbed by antioxidant resveratrol by inducing autophagy and subsequently inhibiting lipid metabolism in oral cancer (108). Hence, SREBP1 is postulated to function as an oncogene, suggesting its potential as a viable treatment target of head and neck cancer.

#### 2.1.5 Increased fatty acids oxidation

Physiologically, fatty acids are catabolized via the mitochondrial fatty acid β-oxidation (FAO) pathway to meet the human daily energy requirements (109). In carcinogenesis and tumor progression, FAO plays a vital role in modulating various malignant behaviors, for example, tumor growth, survival, stemness, drug resistance, and metastasis (110).

##### 2.1.5.1 Carnitine palmitoyl transferase 1

The first and rate-limiting step of FAO is catalyzed by CPT1, comprising three isoforms, CPT1A, CPT1B, and CPT1C (111). CPT1 has been extensively studied in a variety of cancers, and overexpression of CPT1A accelerates tumor development by stimulating FAO in prostatic cancer (112), breast cancer (113), gastric cancer (114), and hepatocellular cancer (115). In addition to its role in FAO, CPT1 plays a pivotal role in a series of signaling pathways to modulate the gene expression, apoptosis, and neovascularization (116). Intriguingly, CPT1 is another molecular linking lipid metabolism and epigenetic modification. CPT1 coimmunoprecipitates with histone deacetylase 1 (HDAC1) in the nuclear extracts from MCF-7 cells, promoting tumor cell proliferation (117). Treatment with HDAC inhibitors decreased the nuclear expression of CPT1 (117), indicating the potential role of CPT1 as a therapeutic target for epigenetic and metabolic interventions in cancer. However, research on CPT1 in head and neck cancer remain limited. According to Cao et al., CPT1A was consistently activated in radioresistant nasopharyngeal carcinoma (NPC) cells, and was positively correlated with the poor overall survival of NPC patients undergoing radiotherapy. In oropharyngeal squamous cell carcinoma, Barros-Filho et al. confirmed that elevated CPT1A expression was associated with poor survival (118). However, in HNSCC, Lin et al. observed no statistically significant correlations between CPT1 expression and patient age, as well as no statistically significant differences in CPT expression between early and late tumor stages (T1/T2 stage vs. T3/T4 stage) (119). Thus, it is imperative to conduct further investigation into the role and associated mechanisms of CPT1 in head and neck cancer, and election bias or other confounding factors should be taken in to consideration.

### 2.2 Alteration of glycerolipid metabolism in head and neck cancer

#### 2.2.1 *De novo* biosynthesis and catabolism of glycerolipid

The synthesis of glycerolipid utilizes fatty acyl-coenzyme A (FA-CoA) and glycerol-3-phosphate (Gly3P) as substrates. FA-CoA condenses with Gly3P via glycerophosphate acyltransferase, leading to the formation of lysophosphatidic acid (LPA). LPA is subsequently converted to phosphatidic acid by 1-acyl-sn-Gly3P acyltransferase (AGPAT), followed by the production of diacylglycerol (DAG) through the functions of phosphatidic acid phosphatase (PAP) or phospholipids (PLs). DAG, in turn, generates TG by activating DAG acyltransferase (DGAT) (120). During the catabolism of neutral TG, adipose triglyceride lipase (ATGL) first converts TG into diacylglycerols (DGs), and then hormone-sensitive lipase (HSL) hydrolyzes DGs to form monoacylglycerols (MGs). Finally, MG lipase (MGL) hydrolyzes MGs and generates fatty acids and glycerol (121).

#### 2.2.2 Altered glycerolipid profile in head and neck cancer

It has been reported that the plasma level of triglyceride is significantly lower in HNC patients (18, 19). Constantly, a notable decrease of triglyceride was revealed in HNSCC and lymph node metastasis tissues by high-resolution magic-angle spinning (HR-MAS) proton NMR spectroscopy (20). Furthermore, the serum level of triglyceride in precancerous oral lesions as well as oral cancer was found to be reduced when compared with healthy controls. Similarly, the plasma level of triglycerides was also decreased in oral cancer patients when compared to pre-cancerous subjects (21).

#### 2.2.3 Dysregulated glycerolipid biosynthesis and catabolism in head and neck cancer

##### 2.2.3.1 Adipose triglyceride lipase

ATGL, also known as phospholipase A2 (PLA_2_), or patatin-like phospholipase domain containing 2 (PNPLA2), functions as a key enzyme hydrolyzing TGs to DAGs. Dysregulated expression of ATGL has been noted in a series of human cancers. The expression of ATGL in lung cancer is enhanced, indicating an unfavorable survival (122). Increased ATGL has been shown to promote the tumorigenesis of colon cancer in an obesity-augmented manner (123). In HNSCC, including oral cancer and tongue cancer, Ramamoorthy et al. reported a significantly increased activity of ATGL (124). Conversely, our research showed that ATGL was downregulated, leading to lipid droplets accumulation in NPC. Furthermore, decreased ATGL was associated with poor prognosis of NPC (125, 126). The variation of ATGL expression might be due to different anatomical regions of the HNSCC. Moreover, the mechanisms underlying the role of ATGL in head and neck cancer need further evaluation.

### 2.3 Alteration of glycerophospholipids metabolism in head and neck cancer

#### 2.3.1 *De novo* biosynthesis and catabolism of glycerophospholipids

The substrate of glycerophospholipid biosynthesis is fatty acyl-CoA, which is obtained via the activation and conversion of fatty acids by acyl-CoA synthase (ACS). The first step in this process is the formation of lysophosphatidic acid (LPA) through the conversion of glycerol-3-phosphate and fatty acyl CoA, catalyzed by glycerol-3-phosphate acyltransferase (GPAT). The second step follows with the formation of phosphatidic acid, catalyzed by lysophosphatidic acid acyltransferase (LPAAT). Additionally, in certain enzyme reactions, different head groups are attached, generating different glycerophospholipids (127). During catabolism, GPLs are transformed into lysophospholipids (LPLs) and free fatty acids by the action of PLA_2_ (128).

#### 2.3.2 Altered glycerophospholipids profile in head and neck cancer

In a study conducted by Silen et al., which involved ten cases of OSCC patients, GPL metabolism was found to be mostly dysregulated, with phosphatidylcholine (PC), phosphatidylethanolamine (PE), and phosphatidylinositols (PI) being the most prominent lipid classes (16). However, in another study by Zhang et al., which included fifty cases of OSCC patients and fifty cases of corresponding healthy controls, all glycerophospholipids were found to be decreased, particularly PC and phosphoethanolamine plasmalogens (22). In addition, LysoPC (14:0) exhibited a stepwise decrease with the development and progression of OSCC (22). Considering the discrepancies between the two studies, further studies should be performed to involve larger sample sizes and proper controls. In the study of Jelonek et al., there was first a significant decrease followed by an increase in the levels of several PCs (PC34, PC36, and PC38 variants) and lysophosphatidylcholine (LPC16 and LPC18 variants) in HNC samples after radiotherapy (RT), in a radiation dose positively pattern (129). The precise changes within the glycerophospholipid profile require additional elucidation, and understanding the specific alteration pattern of these molecules could potentially serve as an indicator for radiotherapy effectiveness.

#### 2.3.3 Dysregulated glycerophospholipids biosynthesis and catabolism in head and neck cancer

The PLA_2_ superfamily, a key group of enzymes involved in glycerophospholipid catabolism, consists of over 30 different types of enzymes. These enzymes are categorized into six subfamilies, including cytosolic PLA_2_s (cPLA_2_s), calcium-independent PLA_2_s (iPLA_2_s), secreted PLA_2_s (sPLA_2_s), lysosomal PLA_2_s, platelet-activating factor (PAF) acetylhydrolases, and adipose specific PLA_2s_ (130, 131). In general, PLA_2s_ are activated and upregulated in several human cancers (132–135). In a study involving five HNSCC patients, the metabolic profile was investigated using ^1^H nuclear magnetic resonance (NMR) spectroscopy. The result revealed a significant elevation in the activity of PLA_2_, especially cPLA_2_, suggesting that PLA_2_ may be a potential anti-cancer target of HNSCC (124). Additionally, in plasma level, Menschikowski et al. reported an increase in secretory phospholipase A_2_ IIA (sPLA_2_-IIA), which was significantly associated with shorter survival among HNC patients (136). Furthermore, Askari et al. observed constant activation of sPLA_2_-IIA in OSCC tissues, and its negative correlation with the level of linoleic acid suggested that sPLA_2_-IIA could serve as a possible indicator of lipid metabolism alteration in OSCC (137). Despite these findings, limited research exists on the regulation of glycerophospholipid synthesis-related enzymes in HNSCC.

### 2.4 Alteration of sphingolipids metabolism in head and neck cancer

Bioactive sphingolipids encompass important lipid molecules, including sphingosine, ceramide, sphingosine-1-phosphate (S1P), and ceramide-1-phosphate. These sphingolipids play a pivotal role in a multiple of biological processes such as cell mobility, proliferation, and survival (138, 139).

#### 2.4.1 *De novo* biosynthesis and catabolism of sphingolipids

Ceramide is a central molecule in sphingolipid metabolism and could be formed by the condensation of serine and palmitoyl coenzyme A. The rate-limiting step in this process is catalyzed by the enzyme serine palmitoyltransferase (SPT) (140). Alternatively, ceramide can be generated by hydrolysis of complex sphingolipids via sphingomyelinase (SMases). Moreover, ceramide can be converted into sphingosine-1-phosphate (S1P) through the action of ceramidases (CDases) and sphingosine kinase 1 and 2 (SphK1and SphK2) (139, 140). Sphingomyelin synthase (SMS) takes phosphatidylcholine (PC) as a donor, inserting the choline group into ceramide as a head group, and converting ceramide to SM. Additionally, ceramide can be transformed into ceramide-1-phosphate (C1P) by the enzyme ceramide kinase (CERK). Furthermore, the metabolism of ceramides also gives rise to complex sphingolipids. As for the catabolism, ceramide is hydrolyzed by CDases, releasing free fatty acids and sphingosine (139, 140).

#### 2.4.2 Altered sphingolipid profile in head and neck cancer

There is limited data available on changes in sphingolipid profiles in HNC. As reported by Ji et al., the concentration levels of sphingolipid 42:2 and 42:3 (SM 42:2 and SM 42:3) were significantly downregulated in laryngeal carcinoma patients when compared to those with laryngeal benign tumors and healthy controls (23).

Ceramide is one of the hub nodes in sphingolipid signaling. As reported by Ogretmen et al., the level of total ceramide was decreased in non-squamous head and neck cancers but increased in HNSCC tumor tissues. Interestingly, the level of C18-ceramide was unexpectedly lower in HNSCC (approximately 50% lower) (24). Increased of C18-ceramide by mammalian upstream regulator of growth and differentiation factor 1 (mUOG1) and mouse homologue of longevity assurance gene 1 (mLAG1), could inhibit the proliferation of HNSCC cells, possibly due to its modulation of telomerase activity and mitochondrial function (24). In accordance with the former study, Senkal et al. observed a low level of C18-ceramide and an upregulated level of C16-ceramide in HNC. Interestingly, C18-ceramide was proposed to have a pro-apoptotic function, whereas C16-ceramide exhibited a protective effect against ER stress and apoptosis (25). Similar decrease in ceramide was observed in laryngeal carcinoma and OSCC when compared with premalignant and normal controls (141–143). Clinically, HNSCC patients with reduced C18-ceramide in tumor tissues tended to have a higher incidence of lymphovascular invasion and lymph node metastasis, which consequently correlated with a higher overall stage of the primary tumor (144). Oppositely, Wang et al. reported that ceramides (d18:1/16:0 and d18:1/18:0) were significantly increased in OSCC patients and positively correlated with pathological stage (22).

Considering the critical functions of ceramide in cancer, more and more research is focusing on its role in cancer therapy. An *in vitro* study showed that ceramide enhanced paclitaxel-mediated apoptosis. A combination of paclitaxel and ceramide could rewire the cell cycle of HNSCC cells, eliminating cells from S and/or G2-M phases, indicating that this combination may offer an attractive alternative to conventional chemotherapy of HNSCC (145). L-reo-C6-Pyridineium-ceramide-bromide (L-t-C6-Pyr-Cer), a cationic water-soluble ceramide analogue, inhibited the growth of HNSCC cell lines with a low IC50. When combined with gemcitabine (GEM), it significantly prevented tumor growth of HNSCC *in vivo*. Moreover, the treatment effect of L-t-C6-Pyr-Cer/GMZ was 2.5 times more effective than that of 5-fluorouracil/cisplatin combination (146). Doxorubicin (DOX), an inducer of ceramide production, increased the level of C18-ceramide, inhibited cell growth, and induced cell death in HNSCC patients when combined with GEM (147). In addition, a phase II clinical study demonstrated that GEM/DOX treatment facilitated the chemotherapeutic efficacy in HNSCC patients who failed the first-line platinum therapy (148).

#### 2.4.3 Dysregulated sphingolipids biosynthesis and catabolism in head and neck cancer

##### 2.4.3.1 Sphingosine kinase 1

SphK1, a key enzyme participating in sphingosine-1-phosphate (S1P) synthesis, has been found to be consistently overexpressed in both HNSCC cell lines and primary tumors (149–151). Its expression was higher in HNSCC cells displaying a more invasive phenotype (152). The upregulation of SphK1 has been correlated with advanced tumor stages, lymph node involvement, recurrence of tumor, and poor survival in HNSCC (149, 150). Mechanistically, SphK1 promotes the invasive ability of human tongue squamous cell carcinoma by upregulating EGFR and STAT3 (152). What’s more, inhibition of SphK1 suppressed cell proliferation and enhanced the radiosensitivity of HNSCC cells (151, 153). In summary, SphK1 might function as an oncogene in HNSCC.

##### 2.4.3.2 Acid ceramidase

Acid ceramidase is responsible for the degradation of ceramide within lysosomes (154). Overexpression of AC has been reported in a variety of human cancers, including HNSCC (155, 156). Interestingly, it is higher in HNSCC cell lines generated from metastasis tumor compared to those from primary tumor (157). As reported by Norris et al., overexpression of AC increased the resistance of HNSCC cells to Fas-induced apoptosis (155). Jang et al. found a negative correlation between AC expression and cisplatin sensitivity in head and neck cancer cells. Treatment with AC inhibitor N-oleoyl-ethanolamine (NOE) or genetic silencing of AC might be novel approaches to enhance cisplatin cytotoxicity (156). Moreover, Movila et al. reported that phosphoethanolamine dihydroceramide (PEDHC) derived from P. gingivalis downregulated the expression of AC, promoted the accumulation of ceramidase and inhibited the proliferation and migration of OSCC cell lines (158). Collectively, overexpression of AC facilitates the malignant behaviors of head and neck cancer cells, and it might be a potential target of head and neck cancer treatment.

### 2.5 Alteration of sterol lipids metabolism in head and neck cancer

Sterol lipids consist of various compounds, including sterols, steroids, secosteroids, bile acids, and others. Among sterols, there are cholesterol, ergosterol, stigmasterol, C24 propylsterol, etc. As the most prominent sterol lipid, cholesterol is widely distributed in mammalian cells. Functionally, sterol lipids are essential for cell membrane formation and participate in a diverse range of physiological and biological processes (159).

#### 2.5.1 *De novo* biosynthesis and catabolism of sterol lipids

Taking cholesterol as an example, it is mainly synthesized via the mevalonate pathway, starting with acetyl-CoA and processing with the involvement of more than 20 enzymes. The major rate-limiting enzyme, 3-hydroxy-3-methylgrutaryl (HMG)–CoA reductase (HMGCR), transforms 3-hydroxy-3-methylgrutaryl (HMG-CoA) into mevalonate. Mevalonate is converted into farnesyl pyrophosphate (FPP), and two molecules of FPP are condensed to yield squalene. Squalene is then oxidized by squalene epoxidase (SQLE) to generate 2,3-epoxysqualene, lanolin alcohol, and cholesterol subsequently (91). Maintaining cellular cholesterol homeostasis is crucial for normal physiological processes. Excessive intracellular cholesterol is exported from cell by ATP-binding cassette (ABC) transporter to reduce intracellular cholesterol levels (160). As for its circulation, the cholesterol synthesized and obtained exogenously which is stored in the liver, is released into the bloodstream in the form of very-low-density lipoproteins (VLDLs). These VLDLs are converted into low-density lipoproteins (LDLs) and taken up by peripheral cells (161). Excessive circulating cholesterol would be transported to lipid-free or lipid-poor apolipoprotein A-I (apoA-I), leading to the production of high-density lipoproteins (HDLs) (162). Moreover, surplus cholesterol could be esterified by acyl coenzyme A-cholesterol acyltransferase (ACAT) to generate cholesteryl esters, which are stored in lipid droplets or circulated as plasma lipoproteins (163).

#### 2.5.2 Dysregulated sterol lipids profile in head and neck cancer

Studies have shown that the serum level of total cholesterol (TC) in OSCC patients is significantly lower than those in healthy controls (21, 26, 27). In eleven HNC patients, Pereira et al. found a significant positive correlation between baseline LDL-cholesterol levels and changes in radiotherapy-induced carotid intima-media thickness, suggesting that LDL-cholesterol might serve as a predictor for RT-induced carotid atherosclerosis in HNC (28). A retrospective cohort study based on 4,575,818 individuals in Korea revealed that high TC and high LDL-cholesterol levels are protective factors and could reduce the risk of HNC (164). Additionally, a prospective analysis based on 474,929 participants from the UK biobank demonstrated a significant U-shaped association between HDL-C and HNC risk in males (29). Based on the 561,388 individuals of the Swedish AMORIS cohort, we found a positive association between blood levels of TC, apoA-I and the risk of HNC. Furthermore, HNSCC patients showed constantly higher levels of TC and apoA-I during the 30 years before diagnosis (30).

#### 2.5.3 Dysregulated sterol lipids biosynthesis related enzymes in head and neck cancer

##### 2.5.3.1 Sterol regulatory element-binding protein 2

As mentioned previously, SREBP2 plays a critical role in selectively modulating the transcription of genes encoding cholesterologenic enzymes (165). However, there’s limited research on SREBP2 in HNC. According to a study by Yang et al., SREBP2 was significantly downregulated in OSCC tissues and cell lines when compared with normal controls. Restoration of SREBP2 could inhibit cell proliferation, migration, invasion, and induce cell apoptosis in OSCC, suggesting its novel role as a tumor suppressor (166). Nevertheless, further research is needed to fully clarify the role of SREBP2 in HNC.

##### 2.5.3.2 3-hydroxy-3-methylglutaryl–CoA reductase

HMGCR is the rate-limiting enzyme in the mevalonate pathway for cholesterol production. Several studies have found that the downregulation of HMGCR is associated with the progression of various tumors (167–169). Interestingly, the expression of HMGCR was found to be elevated in OSCC (170). Furthermore, additional analysis revealed an increased expression of HMGCR in radiation-resistant HNSCC cells (171).

Statin, an HMGCR inhibitor, is noted not only to decrease the level of cholesterol but also reduce the risk of HNSCC. Moreover, statin use has been linked to improved survival in HNSCC patients, particularly in those with HPV-positive tumors (172). In another study, it was proposed that statin use would facilitate the prognosis of HNC, leading to increased overall survival and cancer-specific survival at 2 years (173). What’s more, the application of atorvastatin reduced the rate of cisplatin-induced hearing loss by 19.7% without compromising the effectiveness of cisplatin treatment in HNC patients (174). However, a Mendelian randomization study proposed that the strategy of cholesterol-lowering in oral cancer and oropharyngeal cancer was confounded, warranting further investigation (175).

##### 2.5.3.3 Squalene monooxygenase

SM, which is encoded by the SQLE gene, is the second rate-limiting enzyme in the process of cholesterol synthesis (3). In HNSCC, an elevated level of SQLE expression and gene amplification has been observed, promoting cell proliferation and correlating with the TNM stage of patients (176, 177). Moreover, a high level of SQLE mRNA expression was negatively associated with the survival of HNSCC patients (178). In addition, SQLE plays a role in the tumor microenvironment in HNSCC. It showed a negative correlation with the infiltration of CD8+ T cells, follicular helper T cells, regulatory T cells, and mast cells, while exhibiting a positive correlation with M0 macrophages and resting mast cells (177). Terbinafine, an inhibitor of SQLE clinically used as an antifungal reagent, has recently gained attention for its anti-cancer effects and is being extensively studied (178). In OSCC, terbinafine has been reported to inhibit the proliferation of cancer cells, possibly by suppressing Raf-MEK-ERK and stimulating the p21(cip1) - and p27(kip1) -associated signaling pathways (179, 180). However, further research is needed to fully understand its potential in treating HNSCC. Taken together, SQLE may serve as a novel biomarker for prognosis and a promising drug target for HNSCC.

##### 2.5.3.4 Enhancer of zeste homolog 2

EZH2, a histone methyltransferase, plays a role in modulating endogenous cholesterol synthesis. It is highly expressed in HNSCC, and its upregulation has been found to be correlated with tumor aggressiveness and poor outcomes in HNSCC patients (181, 182). One of the possible mechanisms behind this correlation might lie in the hypermethylation of tumor suppressor genes induced by EZH2 (182). In addition, EZH2-mediated trimethylation modification of histone H3 lysine 27 directly regulates sterol regulatory element binding transcription factor 2 (SREBF2) and its target gene SQLE, which in turn influences the synthesis of endogenous cholesterol in HNSCC. Inhibition of EZH2 strongly activates genes related to cholesterol synthesis and rewrites cholesterol metabolism. As a result, DZNep, an EZH2 inhibitor, showed an impeded function in the proliferation and survival of the human hypopharynx carcinoma cell line FaDu (182). Furthermore, the effects of EZH2 inhibitors have been found to be enhanced following the inhibition of SQLE (178). Nonetheless, further study is needed to fully elucidate the mechanisms.

#### 2.5.4 Dysregulated sterol lipids trafficking in head and neck cancer

##### 2.5.4.1 Low-density lipoproteins receptor

The LDLR is responsible for trafficking of lipoprotein into cells. Increased level of LDLR has been detected in a variety of tumors, including pancreatic cancer (183), glioblastoma cancer (100), and breast cancer (184). However, the level of LDLR in HNSCC remains unreported. Meanwhile, a Mendelian randomization study suggested that LDLR variants decreased the risk of combined oral and oropharyngeal cancer via heritable reduction of LDL-C (175).

### 2.6 Alteration of prenol lipids, polyketides, saccharolipids metabolism in head and neck cancer

There have been no reports on the dysregulation of prenol lipids, polyketides, or saccharolipids in HNSCC to date, making them promising areas for further exploration.

## 3 Relationship between lipid metabolism and risk factors of head and neck cancer

### 3.1 Tobacco and alcohol consumption

Tobacco consumption is a well-established etiological factor in HNC (185, 186). Studies have consistently shown that HNSCC patients with a history of tobacco consumption have a significantly lower level of TC when compared with healthy individuals, especially those without a tobacco consumption history (18, 27, 187, 188). However, there’s no difference in serum levels of TC, LDL, VLDL, HDL, and triglyceride between oral cancer patients with and without tobacco consumption habit (189, 190). Interestingly, the level of serum HDL in oral cancer and oral precancer patients with habit of tobacco was decreased when compared to healthy controls with habit of tobacco use (189). Thus, tobacco carcinogens may increase the generation of free radicals and reactive oxygen species, leading to elevated oxidation/peroxidation rates of polyunsaturated fatty acids, which in turn affect the basic components of cell membranes and potentially be involved in the process of carcinogenesis (191).

Alcohol consumption is another established risk factor of HNC (192, 193). Prediagnosis alcohol intake has been associated with significantly poor overall survival in HNSCC patients in a dose-dependent pattern (194), which might be modified by the genetic polymorphisms of ADH1B and ALDH2 (195). Chronic excessive alcohol consumption is proposed to impair the effect of PPARα, which is involved in β-oxidation, disrupts the biosynthesis of cholesterol, and induces the accumulation of triglycerides (196).

### 3.2 Infection of human papillomaviruses

HPV infection has emerged as a hot spot in HNC research. HNSCC patients with different HPV infection status have different clinical outcomes (197, 198). Two virus oncoproteins, E6 and E7, contribute to the tumorigenic potential of HPV by inhibiting and degrading the tumor suppressor p53 and retinoblastoma-associated protein (pRB), respectively (199). What’s more, E6 stimulates hypoxia-inducible factor 1-α (HIF1α), leading to the activation of SREBP1, which, in turn, increases lipid synthesis by stimulating the expression of FASN and ACC. Additionally, HIF1 could promote the lipid uptake via upregulating receptor proteins from the CD36 family and FABPs (200). Moreover, E6 activates PI3K/Akt/mTOR pathways, resulting in the upregulation of downstream SREBP1 to mediate adipogenesis (201). The inactivation of Rb by E7 also activates the PI3K/Akt/mTOR pathway to promote adipogenesis (202). In HPV-positive HNSCC, a series of genes, such as PIK3CA, DDR2 or NF-kB, are found significantly mutated, contributing to the stimulation of glutamine and lipid metabolism (203–205). Meanwhile, HPV-negative HNSCC often exhibits inactivation of tumor suppressors, for example, p53, leading to the promotion of glycolysis (203–205).

### 3.3 High-fat diet and obesity

Unhealthy diet pattern disturbs the lipid metabolism and plays an important role in HNSCC. The consumption of fried meals, high-fat and processed meats, and sweets has been linked to an increased risk of laryngeal cancer (206–208). Specifically, the consumption of “unsaturated fats” and “animal unsaturated fatty acids” has been identified as risk factors for laryngeal carcinoma but protective factors for oral and pharyngeal cancer, as supported by references (209, 210). Intriguingly, different fat subtypes have been associated with the prognosis of HNSCC patients, impacting outcome factors such as recurrence and mortality. It was shown that high long-chain fatty acid (LCFA), unsaturated fatty acid, ω-3 PUFAs, and ω-6 PUFAs diet was significantly associated with a reduced risk of all-cause mortality, respectively (211). Additionally, a high intake of unsaturated fatty acid has been shown to reduce the specific mortality risk associated with HNSCC (211). Interestingly, it has been observed that a diet pattern with a low ω-6/ω-3 fatty acid ratio (ω-6/ω-3 = 2) suppressed carcinogenesis in the DMBA/BQE-induced hamster oral cancer model via inhibiting the expression of NF-κB p65, PCNA, and cyclin D1 (212).

Obesity, as one of the consequences of high-fat diet, is associated with an elevated incidence and impacts the overall survival of many human cancer types (213–216). In the complicated crosstalk between adipose tissue and cancer cells, mature adipocytes provide adipokines and lipids to cancer cells, while stromal and immune cells within adipose tissue release paracrine factors into the tumor microenvironment. Concurrently, the proliferation of cancer cells drives lipolysis in adipocytes (217). The possible mechanisms underpinning in the obesity-cancer link are proposed as: chronic inflammation in adipose tissue, oxidative stress, interplay between cancer cells and neighboring adipocytes, obesity-induced hypoxia, genetic susceptibility, immune response, and more (215).

However, the impact of obesity on head and neck cancer remains a subject of debate. There are some studies reported no significant association between body mass index (BMI) and the incidence of head and neck cancer (218, 219). In contrast, Dannenberg et al. reported that obesity was an independent risk factor for T1/2N0M0 OSCC patients, leading to poorer progression survival and disease-specific survival (220). In an *in silico* study, a combination of lipid metabolism-related genes, including TGFB1, SPP1, and SERPINE1, was proposed to be potential prognosis markers of OSCC patients (220, 221). Conversely, there are other studies showing that obesity was more likely to be a protective factor against the development of head and neck cancer (222–224). In a retrospective study, Gupta et al. brought out that being obese at the time of diagnosis of HNSCC (including oropharynx cancer, laryngeal carcinoma, and oral cancer patients; 72% of the patients were identified as stage IVA/B, and 28% identified as stage I-III), was an independent prognostic factor conferring better survival, suggesting extended time to recurrence, and better improvement of distant control (223). In a multinational case-control study conducted across nine countries, a low BMI was found to be associated with an increased risk of oral cancer. This conclusion remained consistent even after stratified analyses were performed (224). The underlying factors contributing to this contradiction might lie in the confounding variables such as age, alcohol and cigarette consumption, cancer stage, and treatment characteristics. A systemic review has summarized that obesity mechanically influences the levels and activities of lipid metabolism-related molecules (e.g., FFA, FAS, sPLA_2_, FABP4, and FABP5), thereby contributing to the carcinogenesis and progression of head and neck cancer (225). Nonetheless, the precise mechanism through which obesity may reduce the risk of head and neck cancer remains elusive. In conclusion, further research is required to elucidate the relationship between obesity and head and neck cancer. And of importance, promoting a healthy diet and lifestyle should be emphasized in public health education for cancer prevention.

## 4 Conclusions

**Figure 2:**
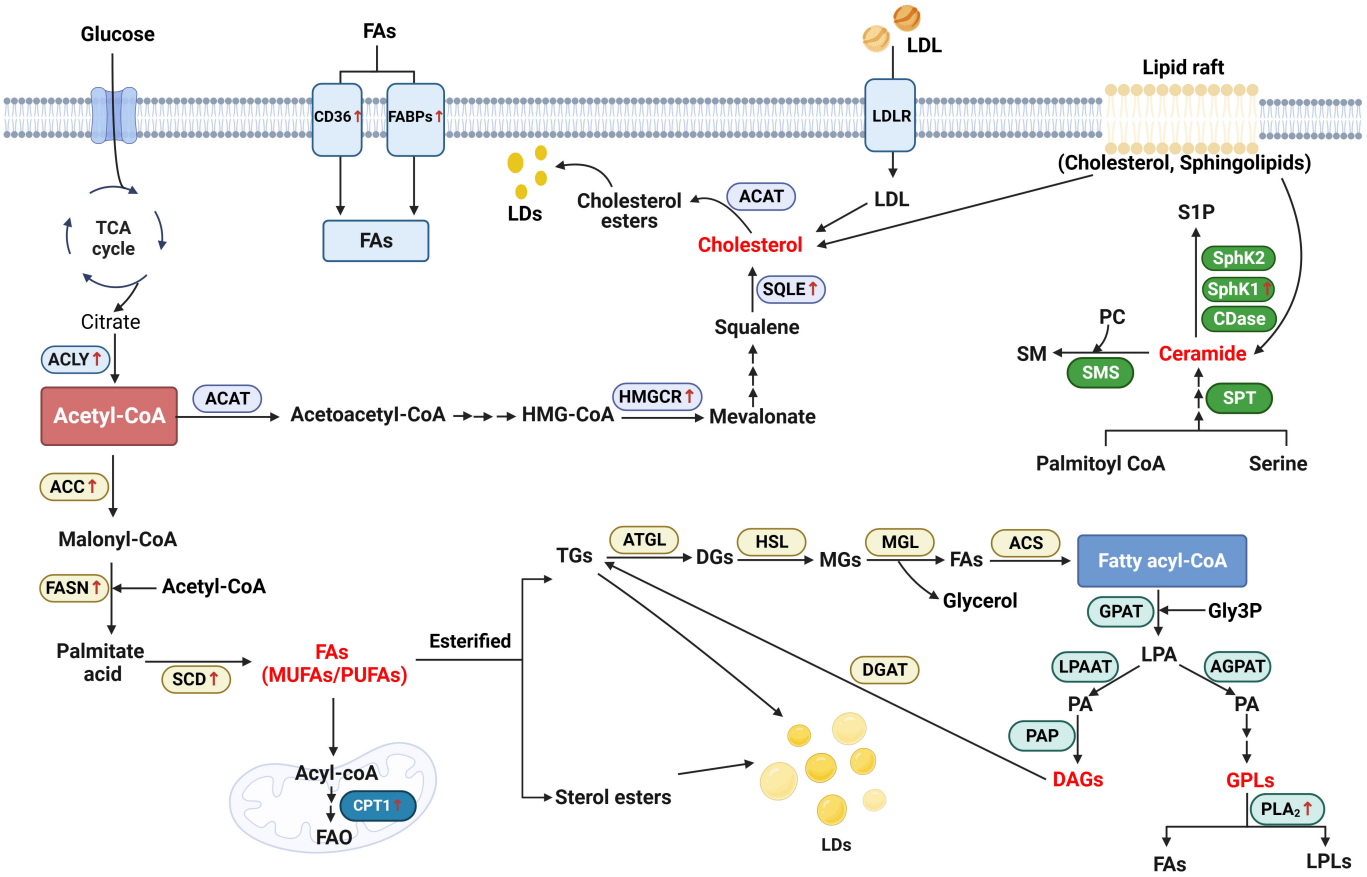
Schematic representation of lipid metabolism reprogramming implicated in head and neck cancer. Generally, in the biosynthesis of fatty acids (FAs), acetyl-CoA generated from citrate is converted to malonyl-CoA by the upregulated acetyl-CoA carboxylase (ACC). Malonyl-CoA combines with acetyl-CoA to generate palmitate acid via activated FASN. The palmitate acid is elongated to form monounsaturated fatty acids (MUFAs) or polyunsaturated fatty acids (PUFAs) by the activated stearoyl-CoA desaturase (SCD). In the catabolism, FAs are transported into the mitochondrial matrix for oxidation by the activated carnitine palmitoyltransferase 1 (CPT1). Additionally, cluster of differentiation 36 (CD36) and fatty acid-binding proteins (FABPs) which are responsible for the trafficking of FAs are notably upregulated in head and neck cancer (HNC). As an important substrate, acetyl-CoA is also converted to acetoacetyl-CoA and HMG-CoA after a serious of reactions. 3-hydroxy-3-methylgrutaryl (HMG-CoA) is transformed to mevalonate by activated 3-hydroxy-3-methylgrutaryl (HMG)–CoA reductase (HMGCR). Squalene is converted by the upregulated squalene epoxidase (SQLE) to form cholesterol, which is another important lipid category. As for the biosynthesis of glycerolipid, fatty acyl-coenzyme A (FA-CoA) condenses with glycerol-3-phosphate (Gly3P) to generate lysophosphatidic acid (LPA) via glycerol-3-phosphate acyltransferase (GPAT). With the function of 1-acyl-sn-Gly3P acyltransferase (AGPAT) and lysophosphatidic acid acyltransferase (LPAAT), LPA is then transformed into phosphatidic acid (PA). Using phosphatidic as the source, glycerophospholipids (GPLs) is generated from after attached with different head groups and it is degraded by phospholipase A2 (PLA2) to generate FAs and lysophospholipids (LPLs). On the other hand, diacylglycerol (DAG) is generated by the function of phosphatidic acid phosphatase (PAP). DAG, in turn, generates triacylglycerol (TG) by activating diglyceride acyltransferase (DGAT). During the catabolism of neutral TG, adipose triglyceride lipase (ATGL) first converts TGs into diacylglycerol (DGs), and then hormone-sensitive lipase (HSL) hydrolyzes DGs to form monoacylglycerols (MGs). Finally, MAG lipase (MGL) hydrolyzes MGs and generates FAs and glycerol. Ceramide is the main type of sphingolipid. It is generated by the condensation of serine and palmitoyl-CoA via the function of serine palmitoyltransferase (SPT). Ceramide is degraded into sphingosine-1-phosphate (S1P), with the function of Ceramidases (CDases), sphingosine kinase 1 (SphK1) and Sphingosine kinase 2 (SphK2).

Lipid metabolism reprogram plays a critical role in the carcinogenesis and progression of head and neck cancer. In the present review, we have summarized the current understanding and advantages regarding the abnormal lipid metabolism profile, novel biomarkers, and possible mechanisms in head and neck cancer, according to the LIPID MAPS Lipid Classification System and cancer risk factors (**Figure 2**). We also discuss and emphasize their potential applications as biomarkers in the diagnosis, treatment and prognosis of head and neck cancer (**Table 2**).

**Table 2:** Potential lipid metabolic-related therapeutic targets in head and neck cancer.

Category	Targeted Gene/enzyme	Function	Expression in HNC	Reagent	Function of the reagent	HNC type	Reference
Fatty acids	ACC	Fatty acid synthesis	Increased	TOFA	ACC inhibitor	HNSCC	Luo (2017) (67)
	FASN	Fatty acid synthesis	Increased	Orlistat	FASN inhibitor	Orthotopic tongue OSCC	Agostini (2014) (82)
				C75	FASN inhibitor	OC	Boelcke (2022) (84)
				TVB-3166	FASN inhibitor	OSCC	Aquino (2020) (85)
	ACLY	Fatty acid synthesis	Increased	BMS303141	ACLY inhibitor	HNSCC	Göttgens (2019) (98)
Glycerolipids	N/A						
Glycerophospholipids	N/A						
Sphingolipids	Ceramide	Increase the level of C18-ceramide	Decreased	Doxorubicin	Inducer of ceramide	HNSCC	Senkal (2007) (147), Saddoughi (2011) (148)
Sterol lipids	Squalene monooxygenase	Cholesterol synthesis	Increased	Terbinafine	SQLE inhibitor	OSCC	Chien (2012) (179), Li (2013) (180)
	EZH2	Cholesterol synthesis.	increased	DZNep	EZH2 inhibitor	HC	Nienstedt (2018) (181)
Prenol lipids	N/A						
Saccharolipids	N/A						
Polyketides	N/A						

ACC, acetyl-CoA carboxylase; FASN, fatty acid synthase; ACLY, ATP citrate lyase; TOFA, 5-tetradecanoxy 2-furanic acid; OSCC, oral squamous cell carcinoma; HNSCC, head and neck squamous cell carcinoma; OC, oral carcinoma; HC, hypopharyngeal carcinoma; EZH2, enhancer of zeste homolog 2; N/A, no report yet.

Although significant advances have been made, as underscored in our review, there are still many unresolved scientific gaps demanding to be addressed. For instance, the roles of HSL, MCL, prenol lipids, polyketides, and saccharolipids in head and neck cancer need to be clarified. A more in-depth exploration of the related mechanisms is essential to shed light on the signaling crosstalk responsible for the alteration of lipid metabolism. The controversial roles of phosphatidylcholine, acetyl-CoA carboxylase, and especially obesity, remain to be elucidated. What’s more, there’s still an urgent need for the identification of novel therapeutic targets related to lipid metabolism.

In conclusion, a more profound understanding of lipid metabolism alterations and the intricacies of associated mechanisms will improve the accuracy of diagnosis, enable the customization of personalized treatments, and fine-tune prognosis strategies for individuals facing head and neck cancer.

## Author contributions

JL: Conceptualization, Writing – original draft. LL: Writing – original draft. LML: Writing – original draft. XZ: Writing – review & editing. ZZ: Writing – review & editing. YH: Writing – review & editing. XX: Conceptualization, Writing – review & editing.
